# Predicting Homogeneous Pilus Structure from Monomeric Data and Sparse Constraints

**DOI:** 10.1155/2015/817134

**Published:** 2015-05-04

**Authors:** Ke Xiao, Chuanjun Shu, Qin Yan, Xiao Sun

**Affiliations:** State Key Laboratory of Bioelectronics, School of Biological Science and Medical Engineering, Southeast University, Nanjing 210096, China

## Abstract

Type IV pili (T4P) and T2SS (Type II Secretion System) pseudopili are filaments extending beyond microbial surfaces, comprising homologous subunits called “pilins.” In this paper, we presented a new approach to predict pseudo atomic models of pili combining ambiguous symmetric constraints with sparse distance information obtained from experiments and based neither on electronic microscope (EM) maps nor on accurate *a priori* symmetric details. The approach was validated by the reconstruction of the gonococcal (GC) pilus from *Neisseria gonorrhoeae*, the type IVb toxin-coregulated pilus (TCP) from *Vibrio cholerae*, and pseudopilus of the pullulanase T2SS (the PulG pilus) from *Klebsiella oxytoca*. In addition, analyses of computational errors showed that subunits should be treated cautiously, as they are slightly flexible and not strictly rigid bodies. A global sampling in a wider range was also implemented and implied that a pilus might have more than one but fewer than many possible intact conformations.

## 1. Introduction

Type IV pili (T4P) and T2SS (Type II Secretion System) pseudopili are thin flexible filaments extending beyond microbial surfaces [1, 2] and are descended from a common ancestor [3]. Pili from different species might have similar quaternary structures, for they are assembled by highly conserved biogenesis machinery, which comprises more than a dozen proteins. These pili are composed of small, initially inner membrane-localized proteins called “pilins,” the conformations of which consist of a highly conserved N-terminal *α*-helix and a relatively less conserved C-terminal globular domain [4]. For their importance in mobility or protein secretion, T4P and T2SS play significant roles in microbial pathogenicity and are of considerable interest as potential targets of drugs or vaccine. Moreover, some special pili contribute to the process of extracellular electron transfer (EET), known as “microbial nanowires,” which inspire research efforts to understand the physicochemical basis for their conductivity [5]. All these researches will benefit if molecular structures of these pili, which might imply the mechanisms of their assembly and functions, are provided. However, difficulties caused by insolubility of subunits, heterogeneous assembly, flexibility, and presence of other surface appendages, such as cytochromes, obstruct researches of T4P and T2SS at atomic resolution.

A traditional way to study pilus structures at atomic resolution would be a combination of high-resolution structures of subunits, examined by X-ray crystallography or Nuclear Magnetic Resonance (NMR) experiments, and low-resolution envelops of pili filaments provided by cryoelectronic microscope (cryo-EM) data, which are obtained from specimens at cryogenic temperatures. To date more than a dozen pilin subunits, or at least pilin fragments, have been determined and archived in the Protein Data Bank (PDB) [6–10], but only one assembled structure of type IV pilus, the* Neisseria gonorrhoeae* (gonococcal or GC) T4P, has been obtained [11]. Meanwhile, several other pilus pseudo atomic models have been proposed, such as the type IVb toxin-coregulated pilus (TCP) from* Vibrio cholerae* [12] and pseudopili of the pullulanase T2SS (the PulG pili) from* Klebsiella oxytoca* that consist essentially of the major pseudopilin subunit PulG [13], which might also imply the difficulty of acquisition of intact pilus filaments structures.

Craig and colleagues [11] obtained the GC pilus structure by combining X-ray crystallography and cryo-EM data. A 2.3 Å resolution pilin crystal structure has been docked into 12.5 Å resolution cryo-EM maps quantitatively, by utilizing iterative helical real space reconstruction [14]. A TCP pseudo atomic structure from* Vibrio cholera* was also proposed by using the same method. The model was modified for several times, according to newly generated DXMS and cryo-EM and negative stain reconstruction [4, 12, 15]. Besides, Campos et al. described a strategy based on helical symmetry from lower resolution EM studies, on conformation restraints validated experimentally and on molecular modeling, and apply it to the PulG pseudopilus [13, 16]. The three pilus models above are all based on EM data of pili filaments and show common helical symmetry.

In this paper, we proposed an alternative approach which is based neither on higher EM maps nor on accurate* a priori* symmetric information. The new strategy enforces symmetry on the conformations among equivalent subunits in the pili assembly and then “guesses” the symmetric details, combining information of single pilin structures and sparse constraints. It is based on two assumptions: (1) T4P and T2SS pili are helically symmetric; (2) there are few differences in structure between pilins packed in crystals and in pili. As all known pilus structures show a common symmetry: a right-handed helix with ~4 subunits per turn, and the pilin subunits have similar non-globular structure which consists of a globular head and a long N-terminal hydrophobic helix, both assumptions seem strong.

This approach, combining distance constraints obtained from a variety of experiments with helical symmetric information, penalizes conformations which include constrained atom pairs that are out of range, reduces the sampling space, and then biases the process of docking efficiently. It involves two steps: a low-resolution step and a high-resolution one. The former narrows down the range of possible symmetric details, while the latter builds and refines full atomic models. A GC pilus structure was reconstructed by using this method, so were the TCP and PulG pilus. Results of the reconstruction verified that the proposed method could recover the structural details of pilus models. This study is a special case of integrating external constraint data with specific prediction methods and could be an efficient way to predict T4P or T2SS pilus structures, by combining with proper restraints.

## 2. Materials and Methods

### 2.1. The Overall Workflow

**Figure 1:**
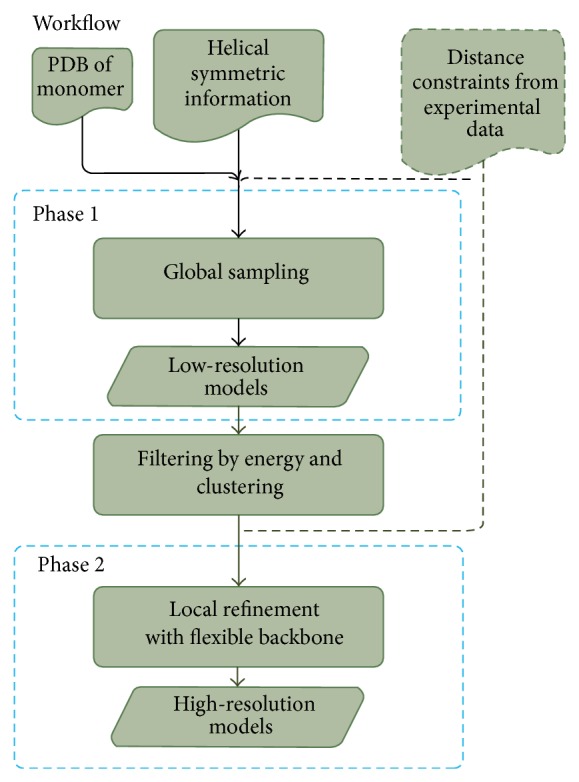
Computational workflow for pilus structure modeling. Distance constraints indicated by dashed lines are optional.

The overall workflow (shown in Figure 1) involves two separated steps: a low-resolution step and a high-resolution one. The first phase aims to find out potential pilus conformations from a wide range of sampling space. It enforces helical symmetry on pilus conformations and generates low-resolution models, or decoys as we call it, by using structures of single subunits, during which the side chains are represented by pseudo atoms. Output models of the global sampling at low resolution could be further analyzed and filtered by their energy scores and clustering results, for the following local refinement in the second step. The second phase carries out local docking perturbations and full atomic refinements around the initial structure and generates high-resolution models. Distance constraints from experiments could be applied to both phases. Moreover, each step could be utilized independently for specific purposes. All the modeling processes are implemented by using the Symmetric Docking protocol in Rosetta software suite [17].

### 2.2. Preparing for Pilin Subunits

Since the GC pilus structure is known as the only structure of intact pilus, the GC pilin subunit was extracted from its pilus structure [11] (PDB ID: 2HIL), with a complete N-terminal *α*-helix and a C-terminal globular domain.

The crystal structure of the TCP pilin TcpA (PDB ID: 1OQV) lacks the 28 amino acid residues in the N-terminal. Because of the fact that TcpA and the* Pseudomonas aeruginosa* PAK pilin (PDB ID: 1OQW) are 75% similar in *α*1N (32% identity) [12], A full length TcpA structure was modeled by employing the coordinates of *α*1N in PAK pilin [4].

The PulG pilin structure derived from crystallography [10] (PDB ID: 1T92) lacks both the C-terminal and N-terminal segments. As proposed by Campos et al. [13] the C-terminal residues were modeled by utilizing the *β*2-*β*3 loop of closely homologous GspG (PDB ID: 3G20) from enterohemorrhagic* Escherichia coli*, and the N-terminal helix, considering its high conservation among T4P and T2SS major pilins, was also built by using the coordinates of *α*1N from PAK pilin.

For the homology modeling, we used MODELLER to reconstruct the C-terminal of the PulG pilin, SWISS-MODEL, and Pymol to build and superimpose the N-terminal *α*-helix. All the pilin models were relaxed by Rosetta Relax protocol to eliminate steric clashes, before the calculations.

### 2.3. Use of Conformational Constraints

#### 2.3.1. Helical Symmetry

Up-to-date data indicate that all known T4P and T2SS pili have similar intact structures with a right-handed helical symmetry along their assembly directions. The GC pilus shows symmetry with a 10.5 Å rise along the symmetric axis and a 100.8° rotation around the axis. Meanwhile, the rise and rotation angle of the TCP are 8.4 Å and 96.7° and 10.4 Å and 84.7° for the PulG pseudopilus.

Taking into account the phenomenon mentioned above, helical symmetry was enforced during all the calculations, which was implemented by defining a symmetrical conformational space through six degrees of freedom (DOF) of rigid-body [17, 18]: the translation along the axis; the rotation around the axis; the distance between the axis and the center of mass (COM) of subunits; and three dimensions of orientation of subunits, that is, *x*, *y*, and *z*. Only one master subunit was taken into real calculation, and all other pilins were just translated from the master through these DOFs.

Considering the fact that the GC pilus structure is the only known full-atom conformation of pilus, an initial helical symmetric definition was extracted from it (shown in Supplementary Material available online at http://dx.doi.org/10.1155/2015/817134) and would be applied to all the following calculations. In addition, initial ranges of the six DOFs could be set to ensure the sampling is taken under some specific situations, for example, specific starting positions and searching ranges.

#### 2.3.2. Distance Constraints

Information from a variety of experimental data could be introduced as distance constraints for our approach and applied to both low- and high-resolution steps. For the three pili discussed in this paper, the distance information was obtained as constrained pairs from either the full atomic structure already known or related experiments, such as cysteine crosslinking, salt bridge charge reversal experiments, and hydrogen/deuterium exchange mass spectrometry (DXMS).

First of all, all the F1/E5 (phenylalanine in position 1 and glutamic acid in position 5) pairs were used as constraints (M1/E5 for TCP), for the simple reason that the proximity of N-terminal nitrogen and Glu5 might be a conserved feature for most T4P and T2SS pili [19] and probably contribute to the stabilization.

Secondly, pairs of residues in adjacent N-terminal *α*-helices were used as constraints to keep the subunits oriented along the axis: for the GC pilus, the pair V9/L16 was determined from the cryo-EM derived model; for the PulG pilus, the pair I10/L16 was from cysteine crosslinking experiments; and for the TCP, a pair of V9/V16 was assumed, as such a distance constraint would help to keep the N-terminal *α*-helices of subunits packed closely in the core of pili.

Besides, other constraints which could be derived from various experiments were also added. For the GC pilus, residue pairs were picked out if they are in close proximity to each other in the intact structure, for instance, several charged residue pairs with the distance between the C*α* atoms less than 10 Å (R30/E49, K76/D153, and K74/E113). Also special atoms pairs, N99/R112, were used as constraints because they are so close in the GC structure and probably form a hydrogen bond to stabilize the whole conformation. For the PulG pilus, restraints derived from cysteine crosslinking and salt bridge charge reversal experiments, or inferred from the proposed structure [13] (R26/L76, R26/E83, K68/I179, V9/V16, and M1/E5), were taken into account. For the TCP, constraints derived from charge reversal experiments and DXMS [15] (R26/L76, R26/E83, and K68/I179) were added.

In addition to the experimental data mentioned above, from which distance constraints came in this paper, other “lightweight,” high-throughput experimental approaches could also potentially yield such constraints [20].

To apply this distance information to the procedures of modeling, distance constraints between pairs of atoms were characterized as energy penalty functions for Rosetta and then energy score penalties would be attached when sampling outside the constraints. All the close distance constraints for the method are set by a flat harmonic function,

(1) fx=0,mmmmmmmmdistance−x0≤tolerance,distance−x0−tolerancesd⁡2,mmmmmmmmmdistance>x0+tolerance,distance−x0+tolerancesd⁡2,mmmmmmmmmdistance<x0−tolerance,

where *x*_0_ represents the center of constraints, which is an estimation of distance between a pair of atoms, tolerance gives the acceptable bound of constraints, and *sd*⁡ stands for standard deviations.

The flat harmonic function guaranteed that models were penalized if the Euclidean distance between two atoms is either too small or too large. For global sampling at low resolution, the three parameters were heuristically chosen as *x*_0_ = 10, tolerance = 5, and *sd*⁡ = 0.5 and the constraints were enforced on C*α* atoms of each residue. For full-atom modeling, the parameters were set as 4, 2, and 0.5, respectively, and the restraints were added on N-O atom pairs in salt bridges [21].

Besides, constraints that define distant relationships from the TCP DXMS were set by bounded constraint function, which describes a linear relationship between the penalty and the distance if it is out of range.

#### 2.3.3. Ambiguous Constraints

Since the arrangement of subunits in the pili remains unknown until their assemblies are determined, it is improper to assign an interaction specifically to two subunits. Ambiguous constraints were therefore used during these calculations. Ambiguous contact between two residues described above could be depicted as an enumeration of all combinations of the residue pairs, respectively, from two different subunits, C1, C2,…, C*n*, and then the ambiguous constraint is described by min⁡⁡(C1, C2,…, C*n*), which picks the minimum from all the scores of possible pairs. Since the total number of subunits was 15 for our calculation, an ambiguous contact should be a combination of 14 possible residue pairs (2∗7, only the master subunit in the middle and the upper 7 subunits are taken into account because of the symmetry). The constraints were implemented by employing Rosetta Constraint Files [22].

### 2.4. Global Sampling

The global sampling phase was used for searching potential pilus conformations from a wide range of sampling space at low resolution, during which the subunits were treated as rigid-body backbones with side chains in centroid mode. A helical symmetry was enforced on the process of sampling as described in last section, and distance constraints were also applied.

Subunits were aligned along the pilus axis to some extent, with the *α*-helix approximately parallel to the axis, in order to optimize the initial position and then accelerate the searching process.

### 2.5. Local Refinement

The local refinement phase, started from a specific initial position, aimed to generate full atomic models with conformational details. A new symmetric definition was generated from the starting point and applied to the calculation with distance constraints being used too. During the local refinement procedure, a small initial perturbation was added to the subunits in low-resolution models first, then side chains were added, by a Monte Carlo Minimization which optimized both the backbones and side chains, and finally, a fast simulated annealing step was employed to relax the full atomic models with flexible backbones.

Command lines for execution of the two steps are shown in Supplemental Material.

### 2.6. Validating and Analyzing the Models

Although a common criterion for judging the results from such calculations is that the best model is with the lowest energy, exceptions are not uncommon during structure modeling. Also, some deviations would be got because of the insufficiency of score functions, the artifacts during data processing, or even the errors from the native structures themselves. To avoid these, we used a combination of clustering and energy score to evaluate our models, as native structure might be situated within a broad basin of low-energy conformations, to keep the efficiency and robustness of structure [23]. Thus, we chose low-energy models from the largest clusters. For low-resolution models, total energy of the master subunits was employed, while for high-resolution models, we also took the interface energy into account as it is an approximation to binding energy [24, 25] and depicts the stability of protein docking.

The pilus structures were clustered based on Root Mean Square Differences (RMSD) of the C*α* positions. A similar strategy of RMSD calculation to the one taken by Campos et al. [16] was used, in which models were rotated around and shifted along their symmetric axes so that the lowest RMSDs could be determined. RMSDs over three consecutive subunits were calculated. Considering that our methods include variables from six degrees of freedom due to rigid-body translations and rotations in addition to differences in the subunit structures, such RMSD could be used to evaluate the accuracy and sufficient to depict structural details of differences among models.

## 3. Results and Discussion

A set of sampling processes have been completed, combining pilin structures with a variety of constraint conditions and searching ranges, shown in Table 1.

For global sampling of the GC pilus at low resolution, 4 different combinations of distance information were applied (lines 1–4, column 3 in Table 1), with 0, 2, 4, and 6 constrained pairs, respectively. The percentage of models in the largest cluster is shown, with symmetric details of the lowest-energy structure from the cluster, as a demonstration. The cutoff of clustering was set to 1.75 Å or 2.5 Å, depending on the constraints and convergent speed. Similarly, low-resolution calculations of the TCP and PulG pilus, with or without constraints, were employed and are shown in Table 1 (lines 5–8), respectively. For each calculation, at least 1000 decoys were sampled and clustered into different groups; only the largest group was taken into account for further processing. To balance the accuracy and computational efficiency, we chose proper low-resolution models from GC3, TC2, and PG2 as initial models for the following high-resolution sampling, that is, GC5, TCP3, and PG3, of which the details are shown in lines 9–11, using a criterion combining energy score and clustering. During each local refinement procedure, at least 1000 models were finally generated.

Specific constraints used in each calculation are described both in Materials and Methods and in Table 1.

### 3.1. Global Searching “Guesses” the Symmetry

As all the known T4P and T2SS pili show right-handed helical symmetry, it is a strong assumption that all T4P and T2SS pili would have similar symmetric modes. In order to narrow the searching space and save computational time, an initial searching range with six DOFs (illustrated in Figure S1) has been set, with rotation angle per unit between 80° and 100°, rise along axis between 5 Å and 15 Å, and COM (center of mass) radius for each subunit between 15 Å and 30 Å. In addition, the orientation of subunits was also perturbed in three dimensions.

**Figure 2:**
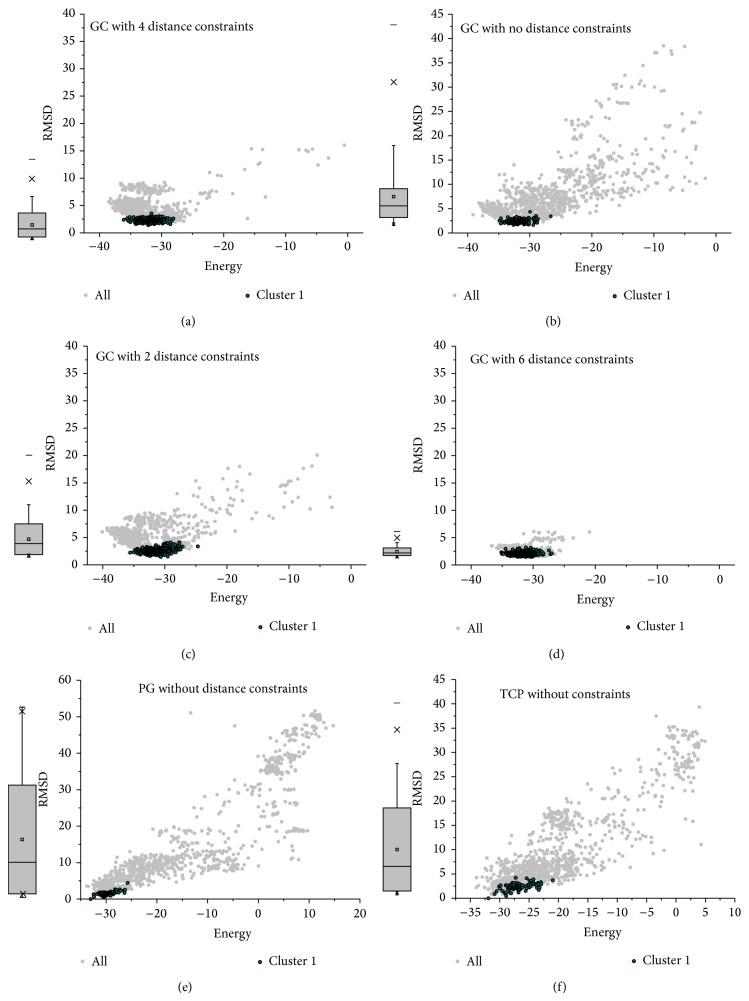
RMSD landscapes from the native structure (for the GC pilus) or the structure with the lowest energy in the largest cluster (for the TCP and PulG pilus) versus energy scores. The grey plots and the boxes show the distributions of RMSDs for all the models from each calculation, and the dark cyan plots show the distribution of cluster 1 (the largest cluster). (a) The GC pilus with 4 distance constraints, (b) the GC pilus with no distance constraints, (c) the GC pilus with 2 distance constraints, (d) the GC pilus with 6 distance constraints, (e) the PulG pseudopilus with no distance constraints, and (f) the TCP with no distance constraints.

It can be inferred that proper distance constraints would narrow down the sampling space and then have a strong influence on the convergence at the largest cluster, as depicted in both Table 1 and Figure 2. Take the GC pilus as example; when with the same cutoff, the number of decoys in the largest cluster increased from 18.10% to 36.00% (Table 1, lines 1 and 2) and from 28.60% to 50.00% (Table 1, lines 3 and 4) after new distance constraints being added. The grey spots in Figures 2(a), 2(b), 2(c), and 2(d) also show the trend that the more the constraints there are, the more convergent the decoys will be. Similarly, data of the TCP and PulG pilus show the same tendency, as the largest cluster of the TCP doubles (Table 1, lines 5 and 6) and the one of the PulG pilus increases from 7.20% to 55.00% (Table 1, lines 7 and 8). Obviously, proper constraints would reduce the sampling space and keep the sampling models “closer” from each other.

The distribution of clusters however shows a quite different tendency against the whole samples. For the GC pilus, no matter how the restraint conditions and the overall trend of distribution change, the cluster seems “stable,” near the native conformation with an average RMSD of approximately 2.5 Å versus the native structure. Even when there were no constraints, decoys tend to converge at clusters near the native conformation, which might imply that information of a single pilin monomer can decide a pilus structure independently, to some extent. This phenomenon can also be found in the TCP and PulG pilus modeling, as shown in Figures 2(e) and 2(f).

As mentioned in Materials and Methods above, we combined energy scores with the results of clustering to pick out proper structures for the following calculations. Also, since the largest cluster is always near the native conformation, the decoys with the lowest-energy score from the largest cluster of each calculation were selected. Their symmetric information has been extracted and shown in the first eight lines of Table 1. The errors are less than 1.5 Å of translation and 2° of rotation angle.

### 3.2. Local Refinement Reveals the Structural Details

**Figure 3:**
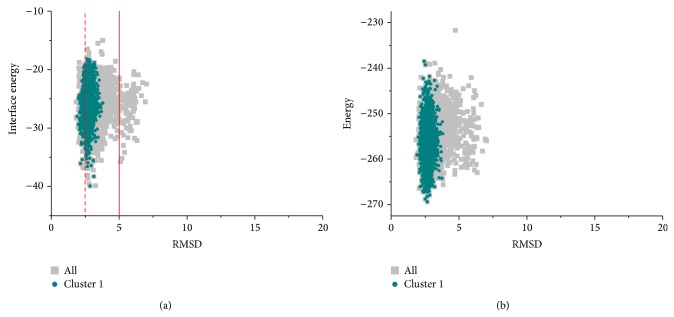
The interface energy and total energy landscapes of full atomic models of the GC pilus versus RMSD from the native conformation. Both show convergence near the native structure.

**Figure 4:**
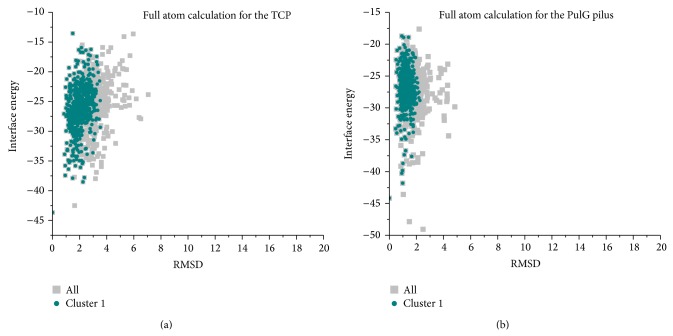
The interface energy landscapes of full atomic models versus RMSD from the lowest-energy conformation in the largest cluster. Left, the TCP. Right, the PulG pilus.

After global sampling within narrow ranges, the lowest-energy decoys among the largest clusters were picked out as the starting conformations for local refinements in high resolution. As described in Materials and Methods, a small initial perturbation was added to the subunits in centroid mode first, then a Monte Carlo Minimization optimized both the backbones and the side chains, and finally a fast relaxation was applied and the backbones of subunits were flexible during the last step. All the three pili were reconstructed, shown in Table 1 (lines 9–11) and Figures 3 and 4. Only for the GC pilus can we compare the results of the procedure with the intact pilus structure obtained from crystallography and EM data. More than 70% of the full atomic models are clustered into the first group, as shown in Figure 3. To distinguish between correct models and incorrect ones, we clustered the results and used both total energy and interface energy score as reference. Figure 3 shows high correlation between the interface energy scores and the RMSDs from the native structure, as the RMSDs of models tend to converge at the point with the lowest score. Besides, most models in the largest cluster are near the lowest-energy model, therefore the native model. Actually, RMSD of the model with the lowest energy is about 2.8 Å from the native one, and its symmetric information is shown in Table 1 (10.97 Å for rise along axis, 100.42° for rotation angle, and 19.16 Å for COM radius). Moreover, most models are clustered between 1 and 5 Å away from the native conformation, with an average of around 2.5 Å, which is exactly similar to the estimated error in atomic position of the native GC pilus model. This accuracy is at the same level as the former method proposed by Campos et al. [16] based on molecular modeling, while the way to take account of RMSD is a little different. Despite the fact that deviations of their models were calculated over all the subunits, our approach takes more degrees of freedom into account, especially the rotation around and the rise along the symmetric axis, as described above. Because of this, our method does not require the detailed symmetric information directly and thus depends less on* a priori* knowledge. In addition, such RMSDs seem sufficient to depict structural details of differences of models.

For the TCP and PulG pilus, due to the absence of published experimentally validated full atomic structures, only the landscape of interface energy versus the lowest-energy model in the largest cluster is depicted in Figure 4. Similar to the GC pilus, most models in the full-atom mode tend to cluster into a large group. The models with the lowest interface energy were taken into account. As shown in Table 1, the TCP model gets a rise of 7.44 Å along the helical axis, 98.72° of the rotation angle around the axis, and a 25.90 Å radius of COM; meanwhile, the symmetric information of the PulG pilus model is 10.75 Å, 86.32°, and 20.03 Å, respectively. Moreover, we compared our TCP model to a pseudo atomic model determined by Craig et al., and the RMSD is about 1.4 Å, which might also validate the reasonableness of our model.

**Figure 5:**
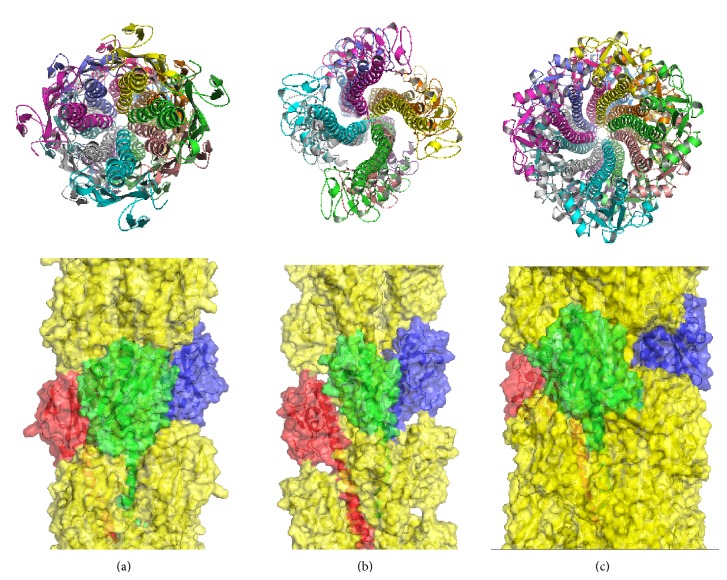
Reconstruction of pili. For structures with the lowest interface energy score from the largest cluster of each pilus, three consecutive monomers are shown in red, green, and blue. Left, the GC pilus. Middle, the PulG pseudopilus. Right, the TCP.

**Figure 6:**
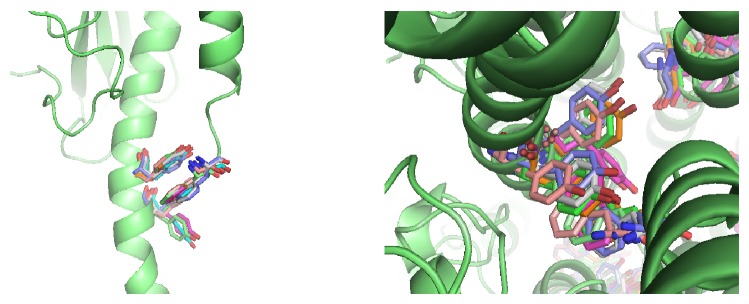
The N-terminal of the GC pilus has three aromatic residues whose side chains are positioned to stack, with F1 from one subunit being inserted between Y24 and Y27 from an adjacent subunit. Aromatic residues from the 10 lowest-energy models are depicted in different colors. Only one backbone of these models is shown (in ribbon).

All the three models show close packing of the pilin subunits, with N-terminal *α*-helices inside the core of pili (Figure 5), which are coincident with former models. Analyses on these structures reveal that such accuracy could recover details which are consistent with experimental phenomena. For example, although there are some deviations between the reconstructed model and the native structure, local details of the structures, such as the aromatic residue stacking of the GC pilus [12], can also be recovered (Figure 6).

### 3.3. Is the Rigid-Body Assumption Strong Enough?

**Figure 7:**
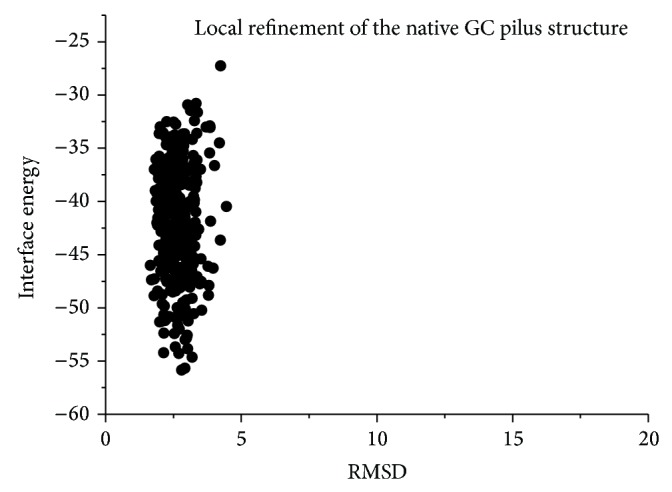
Energy landscapes of local refinement in the native GC pilus structure and the results also show an average deviation around 2.5 Å from the native structure.

In order to figure out where the deviations of models come from, several other calculations were employed. A local refinement of the native GC pilus structure was applied, and the results also show a deviation from the native structure (Figure 7), which is correspondent with the results in the last section. This phenomenon poses a question on whether these RMSDs are derived from artifacts during calculations or from the error of the native structure itself. To eliminate the influence caused by Rosetta energy function artifacts, we used the crystallographic rigid-body transforms to build a repeating lattice [26] out of the model and carried out all-atom refinement in both the lattice and the native symmetric pilus structure.

**Figure 8:**
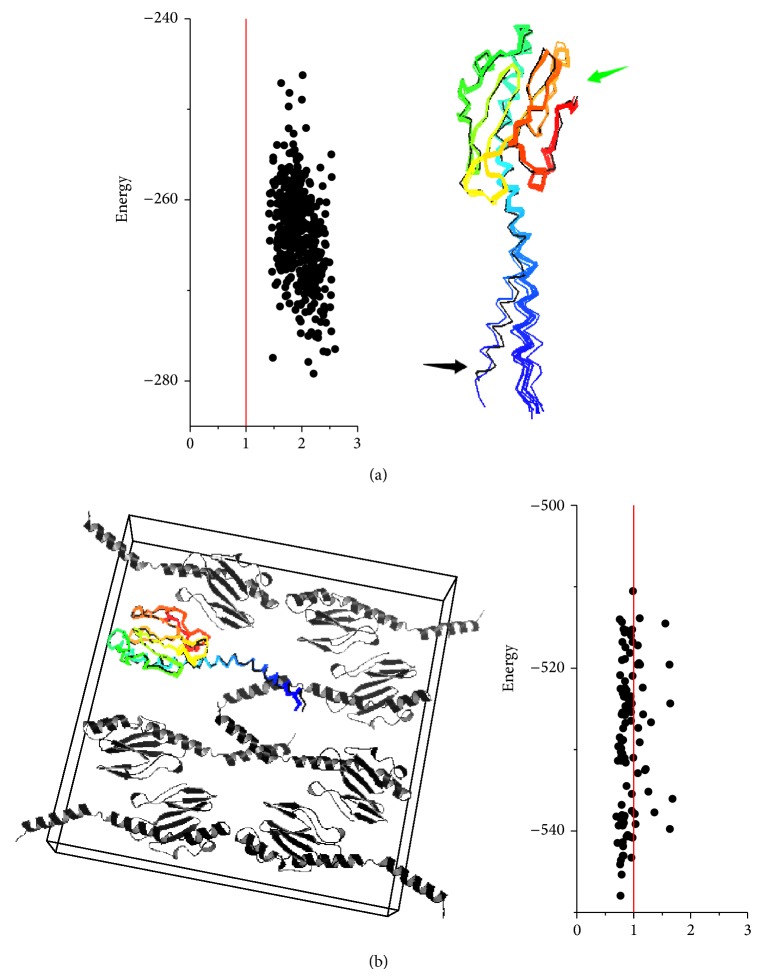
Energy landscapes of the GC pilin in pili versus in unit cells. (a) shows a landscape plot and lowest-energy ensemble for the GC pilin packed in a pilus environment. Evident deviations on the N-terminal helix are shown (indicated by black arrow) between the models (colored) and the native structure (black). (b) shows the same subunits simulated in the crystal environment, including its oligomeric binding partners.

As shown in Figure 8, the models generated from the two procedures exhibit evident differences. RMSDs of the lattice sampling from the native subunit (PDB ID: 2HI2) tend to converge at the point less than 1 Å; by contrast, the pilus sampling exhibits a convergence of RMSD at around 2 Å from the native structure.

To address the difference, we superimposed subunits models into the native structure and found out that evident conformational diversification is shown on the N-terminal *α*-helices while the C-terminal regions remain stable. The analysis confirms that the change of subunit conformation is based on variation of outer environment rather than artifacts derived from the software calculation and also implies that the models we got might have a more reasonable conformation, as our model also has a better MolProbity [27] score than the native one which includes more steric clashes (shown in Table S1).

As mentioned in Introduction section, the current modeling methods of pili, not only the first step of our approach, but also the one which Craig et al. used for the GC pilus [11, 14], are based on the assumption that there are few structural differences between pilins packed in crystals and in pili. Taking into account all the discussion above, this assumption might still be applicable but need to be carefully handled. Considering the rigid-body process is an efficient way to reduce computational complexity, introducing some flexibility during the modeling procedure, or at least parts of the procedure, could be a better choice.

### 3.4. Global Searching in Larger Range Implies More Features of Pili

To get a more complete view of pilus confirmations, global searching with larger ranges has been applied. The initial searching range was set with rotation angle between 0° and 180°, the rise along axis between 5 Å and 15 Å, and the COM (center of mass) radius of each subunit between 15 Å and 30 Å.

**Figure 9:**
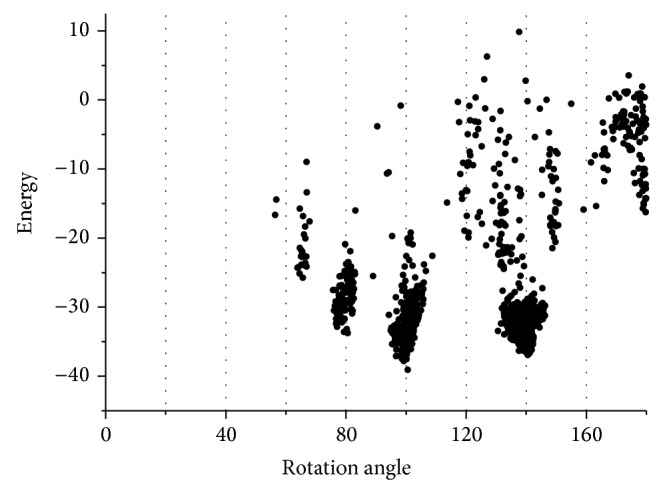
Landscape of energy score versus rotation angle in large range global searching for the GC pilus.

As mentioned in the global searching segment, the structures of the GC pilus tend to converge into a smaller structural space, even with little distance constraints. The results of the large range global searching also support this point (Figure 9). The energy scores have fallen into several troughs at specific rotation angles. Meanwhile, the distributions of diameter and rise are also related with rotation angles, shown in Figure S2, which imply that the initial rotation angle is a key element of the modeling. A similar trend of convergence has also been observed in the sampling of both the TCP and PulG pilus, shown in Figure S3.

All these results indicate that the conformation of pili tends to converge at some specific region, and at least in the common rotation range, which has ~4 subunits per turn, the assembled conformation should be unique for each pilus.

Taking into account the assumption made by Cisneros et al. [28] that the assembly mode and details of major pilin are influenced by some other factors such as minor pilins, it can be implied that, from the perspective of docking energy, a pilus might have more than one possible assembly mode, and if it is true, these assembly modes are limited and influenced by the pilus assembly machinery.

## 4. Conclusions

In this paper, we describe an approach to predict full atomic models of pilus, with sparse constraint data. This method is independent of detailed symmetric data and can “guess” the symmetry from pilin structures and sparse distance constraints which could be obtained from various experiments such as cysteine crosslinking, salt bridge charge reversal experiments, and DXMS. Models of the GC, TCP, and PulG pilus assembly conformations were reconstructed by this method and validated by known structural details of these three pili.

The method combines a low-resolution step with a full atomic one. During the first step, a global searching is performed within a range which contains common helical symmetric information of T4P and T2S pilus. After that, a local refinement is applied in the largest cluster of the first step. The low-resolution models from the first step tend to cluster near the native conformation, and the high-resolution phase, to some extent, can recover the structural details of the native structures.

To assess the quality of models, we use a combination of clustering and energy score to judge output models, as native structure might be situated within a broad basin of low-energy conformations, to fold efficiently and retain robustness to changes in amino acid sequence. The results of the reconstruction of several pili also prove that the criterion is reasonable.

Analyses of the errors for these results show that there are variations of subunits between their conformations packed in crystal and in intact pilus and suggest that we should take a slight flexibility into consideration during the modeling processes, instead of taking pilins as rigid bodies totally.

The global searching in a larger initial range shows that the pilus structures tend to assemble into specific basins, which implies that a pilus may have limited but probably more than one assembly mode, and be influenced by other factors in the pilus assembly machinery, such as minor pilins.

It also can be inferred from this paper that Rosetta could predict the structure of complex macromolecules such as pilus polymers, when with proper constraint information. This method could be a supplement for experimental methods and build pilus models rapidly when without sufficient EM data.

## Supplementary Material

The Supplementary Materials include 2 sets of command lines, a supplementary table and 3 supplementary figures as follows:
Command lines for generating symmetric definition files.
Command lines for execution of the low- and high-resolution steps.
Table S1. Summary of MolProbity scores for both the native structure and the predicted model.
Figure S1. The six DOFs defining the initial searching range.
Figure S2. Distributions of diameter and rise along symmetric axis versus Rotation angle, for the GC pilus.
Figure S3. Landscapes of energy score, rise along symmetric axis and diameter parameters versus rotation angle, for the TCP and the PulG pilus.

## Figures and Tables

**Table 1:** Overview of calculations.

Index^1^	Pilus type	Distance constraints^2^	1st cluster^3^	Detailed symmetric info.^4^				Cutoff^5^ (Å)
				Rise (Å)	Rotation angle (°)	Radius (Å)	Units per turn	
GC1	T4Pa	None	18.10%	11.35	99.32	18.96	3.62	2.50
GC2	T4Pa	F1/E5, N99/R112	36.00%	10.61	100.80	20.28	3.57	2.50
GC3^*^	T4Pa	F1/E5, N99/R112, R30/E49, V9/L16	28.60%	11.58	99.36	18.63	3.62	1.75
GC4	T4Pa	F1/E5, N99/R112, R30/E49, V9/L16, K76/D153, K74/E113	50.00%	11.87	98.62	18.69	3.65	1.75
TCP1	T4Pb	None	10.00%	7.69	100.08	27.88	3.60	2.50
TCP2^*^	T4Pb	R26/L76 R26/E83 K68/I179^§^ V9/V16 M1/E5	21.80%	8.16	98.92	26.08	3.64	2.50
PG1	T2SS	None	7.20%	14.62	76.85	16.08	4.68	2.50
PG2^*^	T2SS	D48/R87, E29/K51, R78/D124, R78/D117, I10/L16, F1/E5	55.00%	10.42	83.00	20.25	4.34	1.75
GC5^¶^	T4Pa	F1/E5, N99/R112, R30/E49, V9/L16,	72.58%	10.97	100.42	19.16	3.59	1.75
TCP3^¶^	T4Pb	R26/L76, R26/E83, K68/I179, V9/V16, M1/E5	60.00%	7.44	98.72	25.90	3.65	2.50
PG3^¶^	T2SS	D48/R87, E29/K51, R78/D124, R78/D117, I10/L16, F1/E5	55.00%	10.75	86.32	20.03	4.17	1.75

^1^Indices of calculations, GC: the GC pilus, TCP: the TCP, PG: the T2SS pseudopilus (PulG pilus).

^2^Distance constraints applied to the calculations, in the form of atom pairs.

^3^Percentage of decoys in the largest cluster.

^4^Detailed symmetric information of picked decoys (with the lowest energy score) from the largest cluster, described by the rise along the axis, the rotation angle, the radius of COM (center of mass) of each subunit, and also the number of subunits per turn.

^5^Cutoffs applied in clustering processes.

^*^Calculation selected for high-resolution sampling in the second step.

^¶^Calculation in high-resolution mode.

^§^Constraint defining a distance no less than 10 Å.
