# Supplementary material for: Predicting Homogeneous Pilus Structure from Monomeric Data and Sparse Constraints
Source: Biomed Res Int. 2015 May 4;2015:817134. doi: 10.1155/2015/817134 (PMC4434193; doi:10.1155/2015/817134)
Supplement: Supplementary file 1 — The Supplementary Materials include 2 sets of command lines, a supplementary table and 3 supplementary figures as follows: Command lines for generating symmetric definition files. Command lines for execution of the low- and high-resolution steps. Table S1. Summary of MolProbity scores for both the native structure and the predicted model. Figure S1. The six DOFs defining the initial searching range. Figure S2. Distributions of diameter and rise along symmetric axis versus Rotation angle, for the GC pilus. Figure S3. Landscapes of energy score, rise along symmetric axis and diameter parameters versus rotation angle, for the TCP and the PulG pilus. [file 817134.f1.pdf]

Supplemental Materials

Command lines for generating symmetric definition files

The symmetric definition files could be obtained from GC pilus structure (PDB ID: 2HIL) by running the make_symmdef_file.pl script:

>perl $rosettadir/rosetta_source/src/apps/public/symmetry/make_symmdef_file.pl -m HELIX -p 2HIL.pdb -r 20.0 -a A -b B -t 7 > symm.def

Additional degrees of freedom (DOF) would be set to make sure the sampling is taken under specific situations, i.e. specific initial positions and searching ranges.

Command lines for execution of the low- and high-resolution steps

Step 1: Global symmetric docking

A pdb file (subunit.pdb) containing the monomeric structure, relaxed and aligned along the helical axis, is used as an input. Constraint file lowres.cst is generated by hand according to the specific constraint pairs.

Execution of the Rosetta procedure:

```
>$rosettadir/main/source/bin/SymDock.linuxgccrelease \
        -in:file:s subunit.pdb \
        -database $rosettadir/main/database \
        -symmetry:symmetry_definition symm.def \
        -packing:ex1 \
        -packing:ex2aro \
        -out:nstruct 1000 \
        -out:pdb \
        -symmetry:initialize_rigid_body_dofs \
        -constraints:cst_file lowres.cst \
        -constraints:cst_weight 10.0 \
        -docking:dock_lowres_filter 1.0 1.0 1.0 \
        -mute core
```

Step 2: Local symmetric refinement

The refinement starting from selected subunit pdbs from step 1. The symmetric definition file symm_highres.def is generated from starting pdbs, named as starting.pdb, using the same perl script mentioned above. An input pdb is aslo generated by this script, as starting_INPUT.pdb. Constraint file highres.cst is generated by hand according to the specific constraint pairs.

Execution of the Rosetta procedure

```
>$rosettadir/main/source/bin/SymDock.linuxgccrelease \
        -in:file:s starting_INPUT.pdb \
        -database $rosettadir/main/database \
        -symmetry:symmetry_definition symm_highres.def \

        -packing:ex1 \
        -packing:ex2aro \
        -out:nstruct 1000 \
        -out:file:fullatom \
        -symmetry:initialize_rigid_body_dofs \
        -constraints:cst_file lowres.cst \
        -constraints:cst_weight 10.0 \
        -constraints:cst_fa_file highres.cst \
        -constraints:cst_fa_weight 100.0 \
```

```
-docking:dock_lowres_filter 1.0 1.0 1.0 \
-mute core \
-docking:kick_relax \
-docking:dock_ppk
```

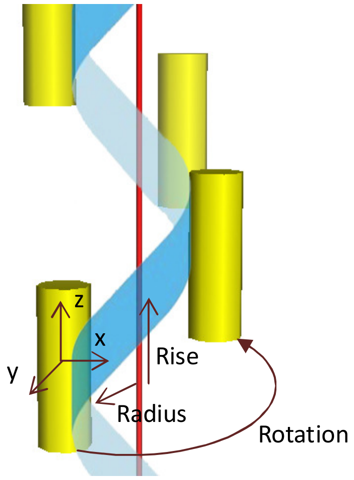

Figure S1. The six DOFs defining the initial searching range

Table S1. Summary of MolProbity scores for both the native structure (PDB ID: 2HIL) and the model we've got with the lowest interface energy.

	GC native structure		Our model		
Clashscore #, all atoms:	107.12		3.88		8th percentile* (N=37, 3Å - 9999Å)
Poor rotamers	288	12.50%	0	0.00%	Goal: <1%
Ramachandran outliers	162	5.77%	45	1.92%	Goal: <0.05%
Ramachandran favored	2484	88.46%	2175	92.95%	Goal: >98%
MolProbity score^	3.92		1.62		38th percentile* (N=342, 3.25Å - 12.75Å)
Cβ deviations >0.25Å	0	0.00%	0	0.00%	Goal: 0
Bad backbone bonds:	18 / 22104	0.08%	15 / 18420	0.08%	Goal: 0%
Bad backbone angles:	18 / 29970	0.06%	15 / 24990	0.06%	Goal: <0.1%

In the two column results, the left column gives the raw count, right column gives the percentage.

* 100th percentile is the best among structures of comparable resolution; 0th percentile is the worst. For clashscore the comparative set of structures was selected in 2004, for MolProbity score in 2006. The specific percentile number in the column is for GC native structure.

#Clashscore is the number of serious steric overlaps (> 0.4 Å) per 1000 atoms.

^ MolProbity score combines the clashscore, rotamer, and Ramachandran evaluations into a single score, normalized to be on the same scale as X-ray resolution.

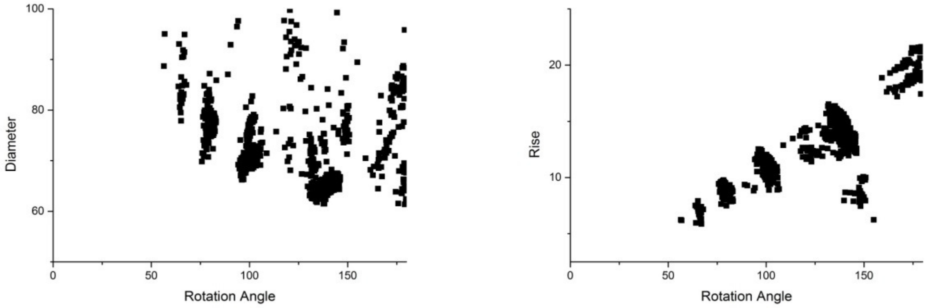

Figure S2. Distributions of diameter and rise along symmetric axis versus Rotation angle, for the GC pilus

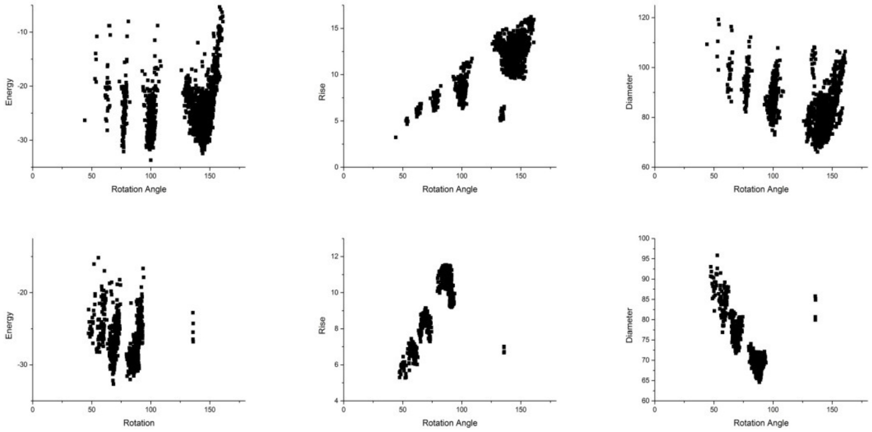

Figure S3. Landscapes of energy score (left), rise along symmetric axis (middle) and diameter (right) parameters versus rotation angle, for the TCP (upper) and the PulG pilus (lower)
